# Deep learning-based spatial analysis on tumor and immune cells of pathology images predicts MIBC prognosis

**DOI:** 10.1371/journal.pone.0328816

**Published:** 2025-08-20

**Authors:** Chao Hu, Fan Wang, Hui Xu, XiQi Dong, XiuJuan Xiong, Yun Zhang, TianCheng Zhao, YuanQiao He, LiBin Deng, XiongBing Lu

**Affiliations:** 1 Department of Urology, The Second Affiliated Hospital of Nanchang University/Nanchang University, Nanchang, PR China; 2 School of Public Health, Jiangxi Medical College, Nanchang University, Jiangxi Provincial Key Laboratory of Disease Prevention and Public Health, Nanchang University, Nanchang, PR China; 3 School of Basic Medical Sciences, Jiangxi Medical College, Nanchang University, Nanchang, PR China; 4 Department of Pathology, The First Hospital of Nanchang City/The Third Affiliated Hospital of Nanchang University, Nanchang, PR China; 5 David Geffen School of Medicine, University of California Los Angeles, Los Angeles, California, United States of America; 6 Department of Jiangxi Province Key Laboratory of Laboratory Animal, Laboratory Animal Science Center of Nanchang University, Nanchang, PR China; Sichuan University West China Medical Center, CHINA

## Abstract

**Objective:**

Muscle-invasive bladder cancer (MIBC) is a highly aggressive disease with a poor prognosis. This study aims to explore the correlation between the spatial distribution of lymphocyte aggregates and the prognosis of MIBC using deep learning.

**Methods:**

Whole-slide images (WSIs) and clinical information of MIBC patients were collected from The Cancer Genome Atlas (TCGA) and the HBlaU079Su01 microarray. After manually annotating tumor and lymphocyte aggregation regions, we developed an automated analysis pipeline, including segmentation and quality control of patches. A convolutional neural network (CNN) classification model was constructed. Based on the definition of the border region of tumor cell nests, we assessed 12 spatial indicators for different patch types within, around and outside the tumor cluster. Using multiomics, we explored the relationships among spatial indicators, clinical factors, the immune microenvironment and molecular mechanisms.

**Results:**

We obtained 301 MIBC WSIs from the TCGA and 43 MIBC WSIs from the HBlaU079Su01 microarray. Two independent CNN models showed excellent classification performance in identifying patches containing tumor cells (AUC = 0.944, 95% CI: 0.942–0.945) and lymphoid aggregates (AUC = 0.934, 95% CI: 0.932–0.937) were constructed. Grad-CAM analysis revealed that some patches contained both tumor cells and lymphocytes. The prognostic value of Lymph_inside % was validated in the HBlaU079Su01 microarray. Multiomics analysis demonstrated that Lymph_inside % demonstrated significant positive correlations with antitumor immune cell abundance and the apoptosis pathway.

**Conclusions:**

Lymph_inside % can be an effective biomarker for predicting MIBC prognosis. This study suggests a novel approach for the development of new prognostic biomarkers based on the spatial distribution of lymphocyte aggregates.

## 1. Introduction

Bladder cancer is the most common type of urothelial malignancy [1]. According to the pathological diagnosis, bladder cancer can be classified into non-muscle-invasive bladder cancer (NMIBC) and muscle-invasive bladder cancer (MIBC). Twenty-five percent of bladder cancers exhibit muscle invasion or metastasis at initial diagnosis, which results in a poor prognosis [2]. Currently, the prognosis of MIBC is primarily predicted based on the TNM stage. By ignoring the heterogeneity of the tumor microenvironment (TME), the predictive sensitivity and accuracy of TNM staging are limited [3,4]. Previous studies have shown that lymphocytes are important targets for antitumor immunotherapy and that their spatial distribution is an important component of heterogeneity in the TME [5,6]. Multiplex antibody (MAB)-based methods can identify cell types and subtypes in the TME and can provide an initial analysis of their spatial relationships and interactions [7]. However, labeling proteins with fluorescence or antibodies is a multi-step process that is time-consuming, experience-dependent, and poorly reproducible.

With the continuous integration of artificial intelligence (AI) technology and medical big data, methods for big data mining based on pathology are being developed rapidly, providing innovative approaches for comprehensively quantifying tumor heterogeneity and achieving precise prognostic prediction for malignant tumors [8]. AbdulJabbar et al reported that quantitative analysis of haematoxylin and eosin (H&E)-stained WSIs can be used to assess the spatial relationship between tumor cells and immune cells and to elucidate the evolutionary trajectory of tumor cells in different immune microenvironments and thus assess the prognosis of non-small cell lung cancer [6]. In addition, predictive models that combine the spatial distribution information of immune cells have also been confirmed to predict the prognosis of liver cancer, breast cancer, and colorectal cancer [9–11]. Therefore, AI-based spatial analysis of lymphocytes in pathological tissues promises to reveal novel prognostic biomarkers for bladder cancer.

The development of AI in pathology has allowed the precise and efficient assessment of immune cell spatial distribution. However, a significant lag has been noted in the development of downstream spatial analysis methods and benchmarking tools. Current tools can perform basic analyses such as the measurement of distances between cell pairs, but current protocols struggle with the diversity of spatial patterns and cannot address the complex biological questions underlying spatial analysis. SPIAT (spatial image analysis of tissues) was developed to promote comprehensive exploration of current and future spatial datasets [12]. This R-based spatial analysis toolkit is compatible with data from any spatial technology and has the potential to transform tissue spatial analysis.

This study integrates novel analytical techniques to establish an automated pipeline that efficiently segments and accurately classifies pathological images, defines border regions of tumor nests, calculates spatial distribution indicators of lymphocyte aggregates, and explores their relationship with MIBC prognosis.

## 2. Methods

### 2.1. Data collection process

Bladder cancer WSIs were downloaded from The Cancer Genome Atlas (TCGA, https://portal.gdc.cancer.gov/). The clinical, genomic, and transcriptomic data required for this study were also obtained from the same database. In addition, bladder cancer WSIs and clinical information obtained from Shanghai Outdo Biotechnology Company (Shanghai, China) served as validation datasets (the HBlaU079Su01 microarray). This study was approved by the Ethics Committee of Shanghai Outdo Biotech Company (Approval number: YBM-05-01) on October 11, 2019. The Ethics Committee waived the requirement for informed consent, as all the samples used in this study were archival paraffin-embedded bladder cancer samples that had been fully anonymized prior to access by the researcher.

Data source: The archived bladder cancer paraffin-embedded samples used in this study were obtained from surgeries performed between May 2007 and November 2011, with follow-up data available until March 2014.

Date of data access: “Data were accessed for research purposes on 15/06/2024.

Access to identifiable information: No identifying information was accessed during or after data collection, and all samples were fully anonymized.

We excluded slides not at 40X magnification, visibly damaged, unclear, or contaminated to ensure consistent image resolution. Only MIBC tumor slides with complete clinical information were included.

### 2.2. Image manual annotation, segmentation, and CNN model construction

Automated image analysis can be affected by staining factors, scanner variability, and imaging artifacts. Robust image preprocessing, normalization, and segmentation methods are essential for achieving clinically satisfactory results [13]. In this study, we implemented automated quality control (QC) to minimize potential biases that might arise from these factors. Fifty WSIs were randomly selected from the TCGA-MIBC dataset. Two pathologists (Xiujuan Xiong and Yun Zhang), following the tumor-infiltrating lymphocytes (TILs) evaluation guidelines for solid tumors [14], used ImageScope software to delineate the “invasive margin” (IM) and to annotate tumor regions and lymphocyte aggregates within the IM. Subsequently, the manually annotated WSIs were segmented into nonoverlapping patches (40 × , 128 × 128 µm) using the OpenSlide library.

After QC, patches were classified according to the pathologists’ annotations. A patch is defined as a “tumor patch” if it contains ≥20 tumor cells, or as a “lymph patch” if it contains ≥20 lymphocytes, with some overlap permitted. All remaining patches are defined as “other patches”. In all, 50,000 tumor patches and 50,000 non-tumor patches were randomly selected. These patches were divided into training and validation sets in an 8:2 ratio to train a convolutional neural network (CNN) model for classifying patches containing tumor cells. The remaining patches were reserved as a test set to evaluate the CNN model’s performance. Model training for identifying lymphocytes followed the same method. Additionally, pathology images from the TMA (n = 43) were manually annotated and segmented and used as an independent external validation set to validate the model.

In this study, we constructed a customized CNN for binary classification based on the VGG-16 architecture. The network accepts RGB images of size (512, 512, [3]) as input. It comprises four convolutional layers (conv2d, conv2d_1, conv2d_2, conv2d_3) and four max-pooling layers (max_pooling2d, max_pooling2d_1, max_pooling2d_2, max_pooling2d_3) for feature extraction. The convolutional layers utilize 3 × 3 kernels, with the number of kernels progressing from 32 to 32, then to 64 and 64. The parameter counts for these convolutional layers are 896, 9,248, 18,496, and 36,928, respectively. Each max-pooling operation reduces the spatial dimensions of the feature maps by half. Subsequent to the convolutional and pooling layers, a Flatten layer is employed to convert the feature maps into a one-dimensional vector. This vector is then passed through two fully connected layers: the dense layer, which has an output dimension of 512 and comprises 33,554,944 parameters, and the dense_1 layer, which serves as the output layer with 2 units for binary classification and contains 1,026 parameters. To mitigate overfitting, a dropout layer is strategically placed between these fully connected layers. The entire model consists of a total of 33,621,538 parameters, all of which are trainable.

### 2.3. Grad-CAM visualization

Gradient-weighted class activation mapping (Grad-CAM) is a technique for interpreting CNNs and visualizing the internal decision-making process of CNN mapping [15]. We employed Grad-CAM to verify whether the model’s identification of tumor and lymphocyte aggregate patches was consistent with the pathologists’ judgements.

### 2.4. Definition of the tumor region and assessment of spatial distribution indicators

SPIAT is an R package for spatial data analysis [12] that can automatically detect tumor cell cluster structure and margins based on tumor cell coordinates, and is used to characterize spatial patterns in tissue images. Following SPIAT’s guidelines, we set min_neighborhood_size = 4 and radius = 2 to define the tumor cell nest border region (including the “Internal margin” and “External margin”). The area inside the IM was divided into “inside” and “outside” regions of the tumor nest.

We calculated the area proportion of the three patch types (tumor, lymph, other) within the IM to create overall indicators: Tumor %, Lymph %, and Other %. We also calculated the proportion of each tissue type in the tumor nest “border” region to generate three spatial distribution indicators: Tumor_border %, Lymph_border %, and Other_border %. Using the same method, we established indicators for the “inside” and “outside” regions, resulting in a total of 12 spatial distribution indicators.

### 2.5. Correlation analysis of Lymph Spatial indicators and Manual TIL Scores

The guidelines for TIL assessment in solid tumors define intratumoral TILs (iTILs) as lymphocytes in direct contact with tumor cells, while TILs located in stromal tissues between tumor cell nests are classified as stromal TILs (sTILs) [14]. In this study, 50 TCGA-MIBC pathology images were randomly selected for both manual TIL scoring and AI-based spatial metric calculations with the aim to investigate the correlation between Lymph_inside% and iTILs%, as well as the relationship between Lymph_outside% and sTILs%.

### 2.6. Relationships between spatial indicators, prognosis, and the TIME

Combined with patient survival information, we analyzed the impact of spatial indicators on patient prognosis. By integrating TCGA multiomics data, we assessed the correlation between spatial indicators and components of the tumor immune microenvironment (TIME). Additionally, pathway analysis was employed to identify molecular mechanisms associated with the spatial indicators.

### 2.7. Statistical methods

The performance of the CNN model was evaluated using receiver operating characteristic (ROC) curves. Survival analysis was conducted with the “survminer” R package, and specifically, its surv_cutpoint function was used to calculate log-rank p-values for different groupings and to select the grouping with the smallest p-value for spatial indicators. Cox regression was used for statistical analysis, with p < 0.05 indicating significance.

## 3. Results

**Fig 1:**
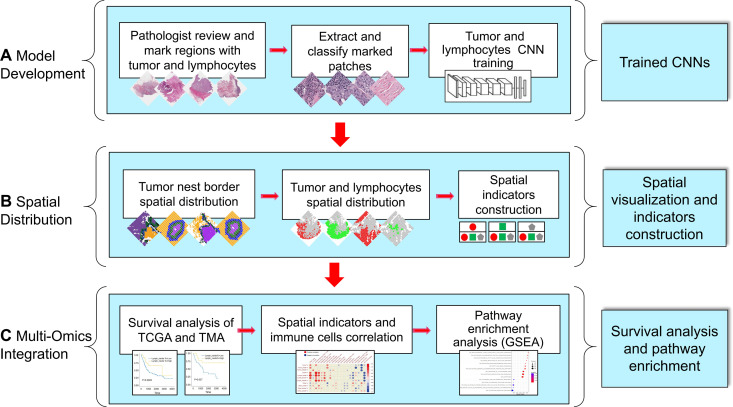
Overview of our workflow. **(A)** CNN models were trained to recognize tumor and lymphocyte aggregate patches. **(B)** Spatial distribution indicators were established. **(C)** The correlations among spatial indicators, prognosis, TIME components, and molecular mechanisms were analyzed.

The workflow of this study is shown in Fig 1.

### 3.1. Training of the CNN model

**Fig 2:**
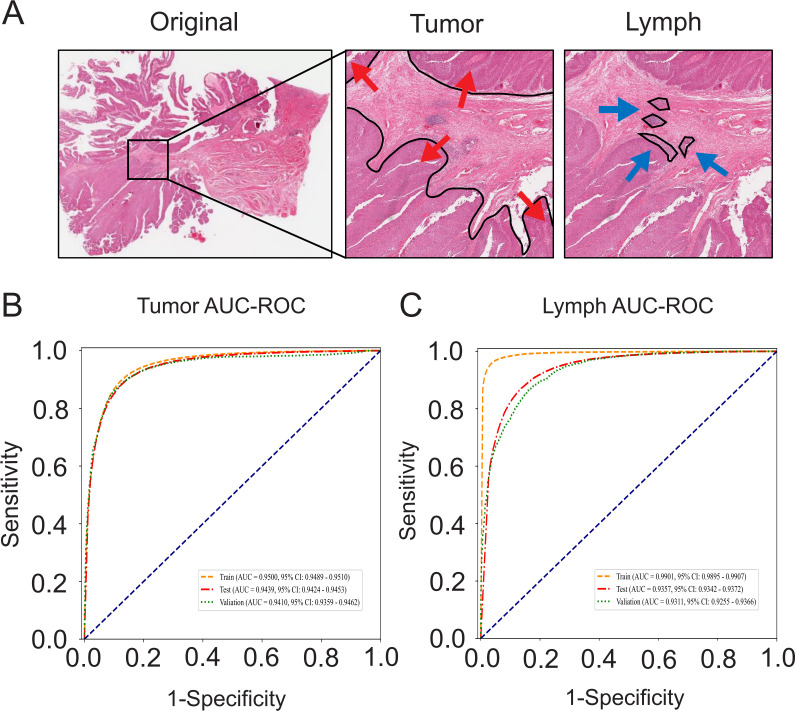
CNN model development. **(A)** Manual annotation results of pathological images, highlighting areas containing tumor cells and lymphocyte aggregates. **(B)** ROC curve of the CNN model for predicting tumor patches (AUC = 0.9439, 95% CI: 0.942-0.945). **(C)** ROC curve of the CNN model for predicting lymphocyte aggregate patches (AUC = 0.9357, 95% CI: 0.934-0.937).

The clinical information is shown in S1 Table. After segmentation and quality control of manually annotated WSIs (Fig 2A), we obtained a total of 543,391 patches. Based on different classification criteria, these patches were divided into 287,353 tumor patches and 256,038 nontumor patches, as well as 155,609 lymphocyte aggregate patches and 387,782 nonlymphocyte aggregate patches (S2 Table). Through training and evaluation, we found that both independent CNN models have excellent classification performance. The tumor-identifying model achieved an AUC of 0.9439 (95% CI 0.9424–0.9453) for the internal test set and 0.9410 (95% CI 0.9359–0.9462) for the external validation set. The lymphocyte-identifying model achieved an AUC of 0.9357 (95% CI 0.9342–0.9372) for the internal test set and 0.9311 (95% CI 0.9255–0.9366) for the external validation set (Fig 2B and 2C).

**Fig 3:**
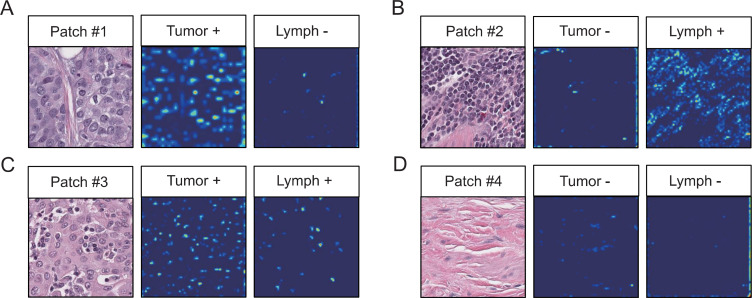
Grad-CAM visualization. **(A)** Tumor patches, highlighting the characteristic area identified by the CNN model as the nucleus of tumor cells. **(B)** Lymphocyte aggregate patches, with the highlighted area of the heatmap indicating lymphocytes. **(C)** Lymphocyte-infiltrated tumor patches. **(D)** Patches without tumor cells or lymphocytes, defined as other patches.

Furthermore, Grad-CAM indicates that the CNN model effectively captures classification features, with the features of tumor cells and lymphocytes concentrated in their respective nuclei (Fig 3). As shown in Fig 3C, we observed lymphocytes infiltrating tumor tissues, further validating the sensitivity and specificity of the models. Nontumor and nonlymphocyte patches are defined as “other” patches (Fig 3D).

### 3.2. Visualization of MIBC tissue spatial distribution

In order to explore the spatial distribution of various patches relative to the tumor cell nest border region, we employed the SPIAT package to delineate the border of the tumor cluster. Notably, the tumor cluster and lymphocyte aggregate regions defined by our model were consistent with the previous manual annotation results.

**Fig 4:**
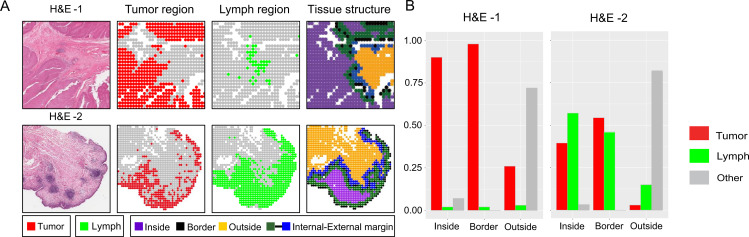
Visualization of tissue spatial distribution. **(A)** In ROI-1, tumor patches and lymphocyte aggregate patches were clearly separated, whereas in ROI-2, there was significant overlap between tumor patches and lymphocyte aggregate patches. **(B)** The proportions of tumor patches, lymphocyte aggregate patches, and other patches in different regions of the tumor cluster are shown in H&E-1 and H&E-2.

Fig 4A shows a clear boundary between the tumor patches and lymphocyte aggregate patches in ROI-1, but ROI-2 shows clear overlap between the two patch types. Additionally, the proportion of lymphocyte aggregate patches in the H&E-2 group was significantly greater than that in the H&E-1 group (Fig 4B). These findings suggest that TME in H&E-2 triggers a stronger immune response.

### 3.3. Correlation analysis of Tumor_inside% with iTILs and Tumor_outside% with sTILs

A correlation analysis with a traditional TIL assessment revealed that Lymph_inside% was highly correlated with iTILs% (R > 0.8) and that Lymph_outside% was highly correlated with sTILs% (R > 0.8) (S1 Fig). This further validated the scientific basis of the spatial analysis of lymphocytes using SPIAT.

### 3.4. Correlations between spatial indicators and clinical factors

**Fig 5:**
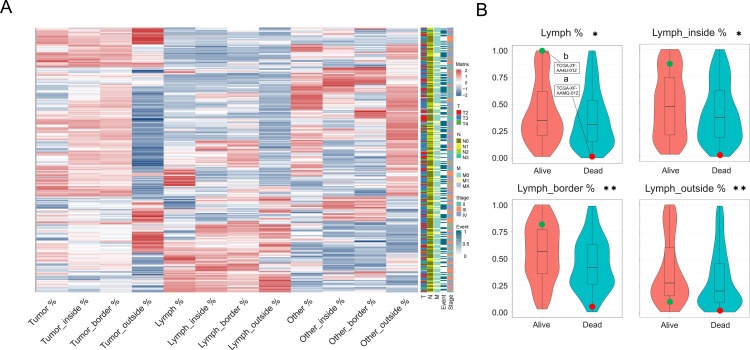
Correlations between spatial indicators and clinical factors. **(A)** Heatmap of 12 spatial indicators in 301 WSIs. **(B)** Distribution of lymphocyte-related indicators under different survival states (*P* value: ✱ < 0.05, ✱✱ < 0.01).

In order to explore the correlation between spatial indicators and various clinical parameters (survival status, TNM stage, and pathological grade), we integrated data from the TCGA (Fig 5A; S2 Fig). Significant differences were observed in Lymph % and Lymph_inside % in tissues from patients with different vital statuses (*P* < 0.05), and highly significant differences were observed for Lymph_border % and Lymph_outside % (*P* < 0.05; Fig 5B). Interestingly, the two patients previously used to demonstrate spatial distribution had different prognoses.

### 3.5. Relationship between spatial indicators and prognosis in TCGA datasets

**Fig 6:**
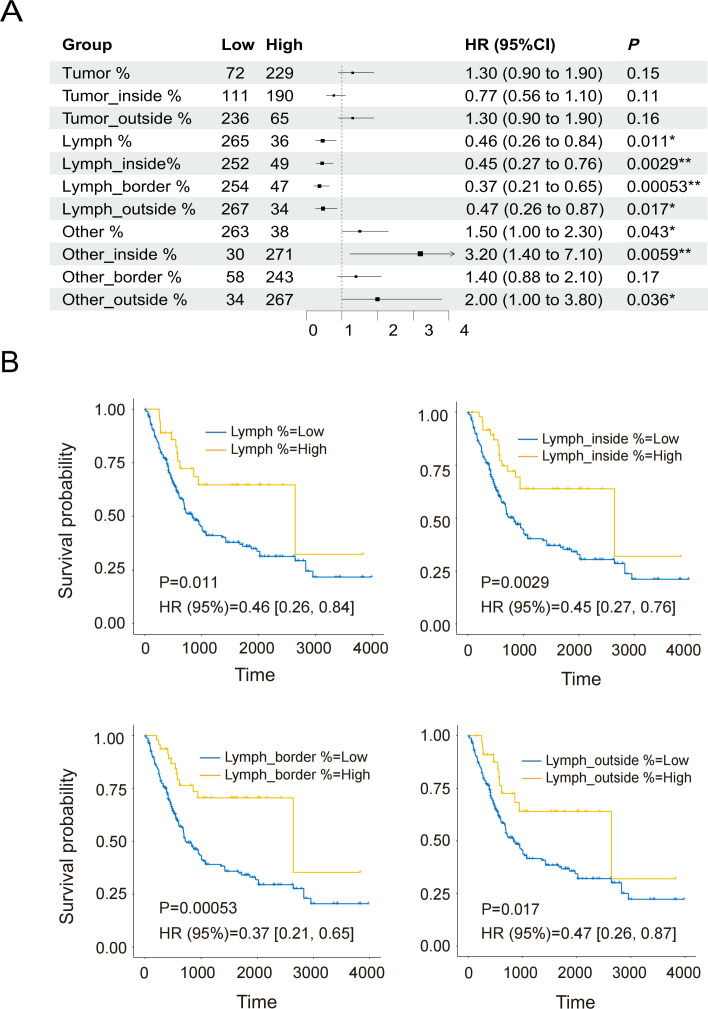
Relationships between spatial indicators and prognosis (TCGA). **(A)** Summary of univariate Cox analysis. **(B)** Kaplan–Meier (KM) curves showing the impact of Lymph % (HR [95% CI] = 0.46 [0.26-0.84], P = 0.011), Lymph_inside % (HR [95% CI] =0.45 [0.27-0.76], P = 0.0029), Lymph_border % (HR [95% CI] = 0.37 [0.21-0.65], P = 0.00053), and Lymph_outside % (HR [95% CI] = 0.47 [0.26-0.87], P = 0.017) on patient survival (*P*: * < 0.05, ** < 0.01).

We performed univariate Cox regression analysis to evaluate the impact of spatial indicators on prognosis. The results revealed that Lymph %, Lymph_inside %, Lymph_border %, and Lymph_outside % were significantly positively correlated with prognosis, whereas Other %, Other_inside %, and Other_outside % were significantly negatively correlated with prognosis (*P* < 0.05; Fig 6A).

Additionally, the survival rates of the high-level groups for lymph-related spatial indicators were significantly greater than those of the low-level groups (Lymph%: P = 0.011, Lymph_inside%: P = 0.0029, Lymph_border%: P = 0.00053, Lymph_outside%: P = 0.017) (Fig 6B). Conversely, the survival rates of the high-level groups for Other-related indicators were significantly lower than those of the low-level groups (Other%: P = 0.043, Other_inside%: P = 0.0059, Other_outside%: P = 0.036) (S3 Fig). To further assess the independent prognostic significance of spatial indicators, multivariable Cox regression analysis was conducted, adjusting for potential confounders such as age, gender, and tumor stage. The analysis revealed that Lymph_inside%, Other%, and Other_inside% are independent prognostic factors for patients. Lymph_inside% remained significantly positively correlated with prognosis (P = 0.028), while Other% (P = 0.034) and Other_inside% (P = 0.040) showed significant negative correlations with prognosis (S3 Table).

### 3.6. Relationships between spatial distribution indicators and prognosis in the TMA dataset

**Fig 7:**
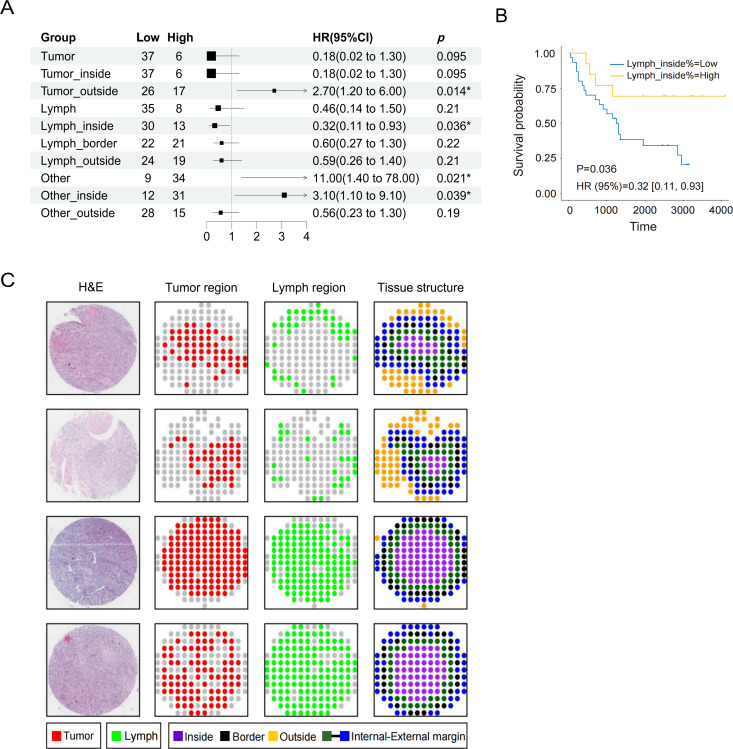
Relationships between spatial indicators and prognosis (HBlaU079Su01). **(A)** Results of univariate Cox analysis. **(B)** KM curve showing the impact of the Lymph_inside % on patient survival (P: ✱ < 0.05, ✱✱ < 0.01). **(C)** Spatial diagrams of patients based on different prognoses.

We calculated the spatial indicators of 43 samples from the HBlaU079Su01 dataset (S4 Table), and the univariate Cox regression analysis indicated that Lymph_inside % was significantly positively correlated with prognosis (*P* < 0.05; Fig 7A). However, the Tumor_outside %, Other %, and Other_inside % were significantly negatively correlated with prognosis (P < 0.05). Kaplan-Meier curves confirmed that patients with high Lymph_inside % had better survival (HR:0.32, 95% CI:0.1–0.93, P = 0.036) (Fig 7B), while high Tumor_outside % (HR:2.70, 95% CI:1.20–6.00, P = 0.014), Other % (HR:11.00, 95% CI:1.40–78.00, P = 0.021), and Other_inside % (HR:3.10, 95% CI:1.10–9.10, P = 0.039) were associated with worse survival (S4 Fig).

Next, we generated spatial distribution diagrams of four patients (Fig 7C). Patients with a poor prognosis had a low proportion of lymphocytes infiltrating the tumor cluster (1 and 2), while patients with a good prognosis exhibited the opposite pattern (3 and 4). On the other hand, the diagrams indicated that most tissues lacked a complete boundary of the tumor area.

### 3.7. Correlations between spatial indicators and components of the TIME

**Fig 8:**
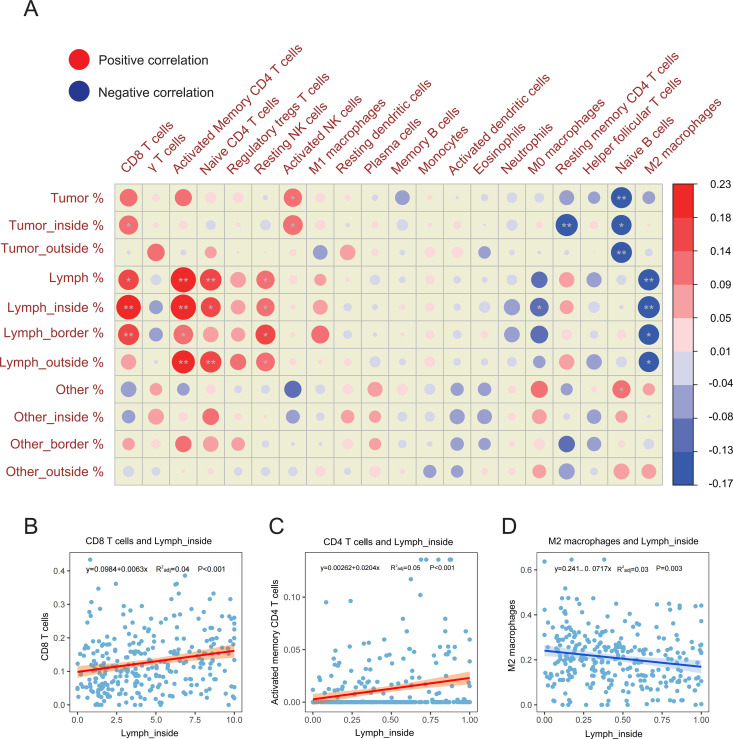
Correlations between spatial indicators and the proportions of immune cells in the TIME. **(A)** Correlations between spatial indicators and the proportions of immune cells within the microenvironment. **(B-D)** Scatter diagrams showing the correlation between Lymph_inside % and the proportions of CD8 + T cells, activated memory CD4 + T cells, and M2 macrophages. The size of the dots is proportional to the Spearman correlation coefficient. Bilateral Spearman correlation analysis was performed using multiple test-corrected P values (*P*: ✱ < 0.05, ✱✱ < 0.01).

The spatial infiltration pattern of immune cells reflects the state of the TME and has been reported to be related to the response to immunotherapy [16]. Therefore, we investigated the relationship between spatial indicators and the abundance of immune cells in TME. As shown in Fig 8A, lymphocyte-related indicators, such as CD8 + T cells and activated memory CD4 + T cells, were significantly positively correlated with the abundance of activated immune cell subsets (*P* < 0.05) and significantly negatively correlated with the abundance of M2 macrophages (*P* < 0.05). Additionally, we plotted scatter diagrams showing the correlations among Lymph_inside % and the proportions of CD8 + T cells, activated memory CD4 + T cells, and M2 macrophages (P < 0.005) (Fig 8B–8D). In contrast, tumor-related indicators were positively correlated with the proportion of CD8 + T cells and activated NK cells (*P* < 0.05) and were significantly negatively correlated with the proportions of resting memory CD4 + T cells and naive B cells (*P* < 0.05).

### 3.8. Relationship between the Lymph_inside % and pathways

**Fig 9:**
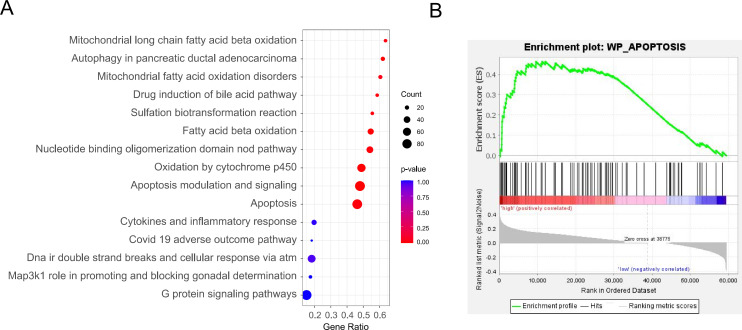
KEGG pathways related to Lymph_inside %. **(A)** Pathways significantly enriched in the group with high Lymph_inside %. **(B)** The GSEA enrichment plot shows that the genes in the apoptosis pathway are among the most significantly enriched.

In order to explore the potential molecular mechanisms of lymphocyte aggregation within the tumor area, we divided patients into high- and low-level groups based on the Lymph_inside % and performed differential gene expression analysis. We obtained MIBC RNA sequencing data from TCGA and used GSEA software for pathway enrichment analysis. As shown in Fig 9A, Lymph_inside % was significantly correlated with the enrichment of KEGG pathways related to processes such as apoptosis, autophagy, fatty acid metabolism, the inflammatory response, and biotransformation (*P* < 0.05). Fig 9B shows the results of the correlation analysis of the Lymph_inside % versus apoptosis pathway enrichment. The enrichment curves indicate that apoptosis-related genes were significantly enriched in the high Lymph_inside % group.

## 4. Discussion

Recent studies have recommended the incorporation of TIL scoring into routine histopathological reporting as a crucial supplementary biomarker for the prognostic evaluation of solid tumors [17–20]. However, manual TIL quantification is associated with significant limitations, including high operational costs, technical complexity, and susceptibility to subjective biases. The advancement of AI in pathology holds promise for the precise quantification of the intricate spatial patterns of TILs within the TME. Leveraging H&E-stained histopathological images from the TCGA, Saltz et al. [21] pioneered the precise generation of TIL maps across 13 cancer types, including bladder urothelial carcinoma, through AI-powered “computational staining”. In-depth analyses have demonstrated the differential enrichment of TIL density and spatial architecture across tumor types, immune subtypes, and molecular subtypes. This suggests that spatial infiltration patterns of immune cells may reflect specific neoplastic aberrations and represent critical manifestations of TME heterogeneity.

Current research predominantly utilizes the presence of sTILs as a prognostic biomarker due to high interobserver reproducibility, while iTILs are frequently overlooked due to their low abundance and marked spatial heterogeneity [22]. The advancement of spatial immune infiltration analysis technologies is fundamentally reshaping our understanding of the TIME, which has necessitated the development of novel analytical paradigms to address the demand for comprehensive immune profiling. In this study, we established two independent CNN-based models to resolve cellular recognition challenges stemming from spatial overlap between tumor cells and lymphocytes within tumor nests, thereby enhancing the evaluability of intranest lymphoid infiltration.

Traditional methods for describing the relative position of lymphocytes compared with the tumor nest margin often rely on manual delineation of the tumor nest margin or annotated machine learning training sets, which can be time-consuming and prone to bias. Furthermore, with the global promotion of standardized evaluation guidelines for sTILs in breast cancer, the definition of tumor stroma composition has exhibited variability due to widely separated tumor cell nests, which has been a key focus [23]. SPIAT is a platform-independent, user-friendly analysis toolkit that can automatically detect and identify tumor nest margin regions, including both the inner and outer margins, based on tumor cell coordinates. In addition to analyzing the tumor nest boundary as a separate region, the spatial indicators Lymph_inside% and Lymph_outside%, constructed based on SPIAT, are similar in definition to the traditional iTILs% and sTILs%, respectively. A correlation analysis has confirmed our hypothesis.

In the TCGA-BLCA cohort, we found that Lymph %, Lymph_inside %, Lymph_border %, and Lymph_outside % were significantly positively correlated with prognosis. However, in the HBlaU079Su01 dataset, only Lymph_inside % was validated as a protective factor. We found that the small area of TMA lacked complete boundaries of the tumor cluster, which limited the comprehensive analysis of lymphocyte distribution. These results could serve as valuable references for the production of optimized pathological microarrays in the future.

Tertiary lymphoid structures (TLSs) are lymphoid-like tissues formed by the aggregation of diverse immune cells (and include B-cell and T-cell zones) in non-lymphoid tissues under pathological conditions such as in tumors and inflammation. TLSs are associated with patient prognosis and treatment response [24–26]. To precisely evaluate the spatial distribution of TLSs, Suzuki et al. [27] used AI to identify TLSs and tumor regions in endometrial cancer, classifying TLSs as proximal (< 500 µm from the invasive margin, IM) or distal (500–5000 µm from the IM). Their research showed that patients with distal TLSs (dTLSs) had significantly longer overall survival and progression-free survival (PFS). Additionally, another study [28] developed a TLS scoring system for head and neck squamous cell carcinoma (HNSCC) by integrating TLS maturation and distribution. They found that patients with mature intra-tumor TLSs (Intra-TLSs) or TLSs at the invasive margin (Invas-TLSs) had higher 5-year survival rates, while those with mature peritumoral TLSs (Peri-TLSs) had poorer prognoses. As the structural basis of TLS formation, the directed aggregation of lymphocytes is crucial. In this study, the spatial analysis of lymphocyte aggregates based on tumor cell nest margins represents an important advancement in lymphocyte spatial analysis.

Different types of cells exhibit complex interactions in the TME. CD8 + T and NK cells can attract tumor cells, promoting the secretion of cytokines such as IL-2, IL-12, and IFN-γ and inflammatory responses [29,30]. CD8 + T cells and activated memory CD4 + T cells are the main executors of antitumor immune responses and can recognize and kill tumor cells [30]. M2 macrophages, cellular immune components of the TME, usually secrete immunosuppressive cytokines such as IL-10 and TGF-β, which promote tumor growth and immune escape [31–33]. In addition, tumor cells evade immune surveillance through various mechanisms, including suppression of naive B cell differentiation and activation of resting memory CD4 + T cells [34,35]. In this study, we found that lymph-related indicators were positively correlated with the proportions of CD8 + T cells and activated memory CD4 + T cells and negatively correlated with the proportion of M2 macrophages. These results suggest that a high proportion of lymphocytes can change the TME, enhancing immune cell infiltration and function.

Next, we identified that the apoptosis pathway is related to the Lymph_inside % indicator. In the apoptosis pathway, highly expressed genes, such as FADD, IRF1/2, NF-KB1, and CASP8, are associated with antitumor immune therapy. Fas-associated death domain protein (FADD) is involved in T-cell receptor signal transduction, promoting the activation and proliferation of T cells [36]. The IRF gene family is crucial for tumor occurrence and immunity. CD8 + T cells significantly infiltrated tumors with high IRF expression scores, while immunosuppressive cells, such as M2 macrophages, were enriched in tumors with low IRF expression scores. The IRF expression score can be a key predictive indicator for prognosis and immune therapy response across cancers [37]. Nuclear factor kappa light chain enhancer of activated B cells (NF-κB) is a key transcription factor complex. Recent studies have shown that the NF-κB pathway can effectively facilitate the recruitment and activation of antitumor CD8 + T cells and is a key factor in prognosis [38]. The high expression of these genes in the apoptosis pathway highlights the potential application of Lymph_inside % in antitumor immune therapy.

In contrast, the stromal cells of the TME also play an important role in the regulation of tumor occurrence and development [39]. Patches of the “other” type mainly included fibroblasts, endothelial cells, and the extracellular matrix (ECM). Fibroblasts can be activated by malignant cells to become cancer-associated fibroblasts (CAFs) [40]. CAFs can affect the infiltration of T cells and the polarization of macrophages in the bladder cancer TME, thereby affecting the response to immunotherapy [41]. In addition, CAFs also affect the infiltration of lymphocytes by reshaping the extracellular matrix [42]. Endothelial cells promote tumor-associated macrophage (TAM) migration and infiltration by expressing adhesion molecules, and they change blood vessel structures to prevent effector T-cell infiltration [43–45]. In summary, the negative impact of Other % on prognosis may be related to the immunosuppressive effects of CAFs and endothelial cells in the TME.

The heterogeneity of the TME leads to significant differences in tumor progression among individuals [46]. Future research will focus on exploring the spatial distribution of stromal cells within the TME. For example, by integrating advanced single-cell and spatial transcriptomics techniques, we aimed to localize CAFs and assess the specific impact of the spatial distribution of their subtypes on prognosis.

## 5. Conclusion

In this study, we successfully developed an AI-based automated pipeline for evaluating spatial distribution and identified Lymph_inside % as an effective indicator for assessing the prognosis of MIBC. This research is expected to provide a robust scientific foundation and technical support for the advancement of precision medicine.

## Supporting information

S1 Fig Correlation between AI and Manual TIL estimate. (PDF)

S2 Fig Correlation between spatial indicators and clinical factors. (PDF)

S3 Fig Correlation between spatial indicators and patient survival (TCGA). (PDF)

S4 Fig Correlation between spatial indicators and patient survival (TMA). (PDF)

S1 Table Clinical and histopathological characteristics. (PDF)

S2 Table Patches data of CNN training. (PDF)

S3 Table Multivariable Cox regression analysis of prognosis. (PDF)

S4 Table Patches data of TMA. (PDF)
