# Supplementary figures and images for: Deep learning-based spatial analysis on tumor and immune cells of pathology images predicts MIBC prognosis
Source: PLoS One. 2025 Aug 20;20(8):e0328816. doi: 10.1371/journal.pone.0328816 (PMC12367112; doi:10.1371/journal.pone.0328816)

# S1 Fig. Correlation between AI and Manual TIL estimate

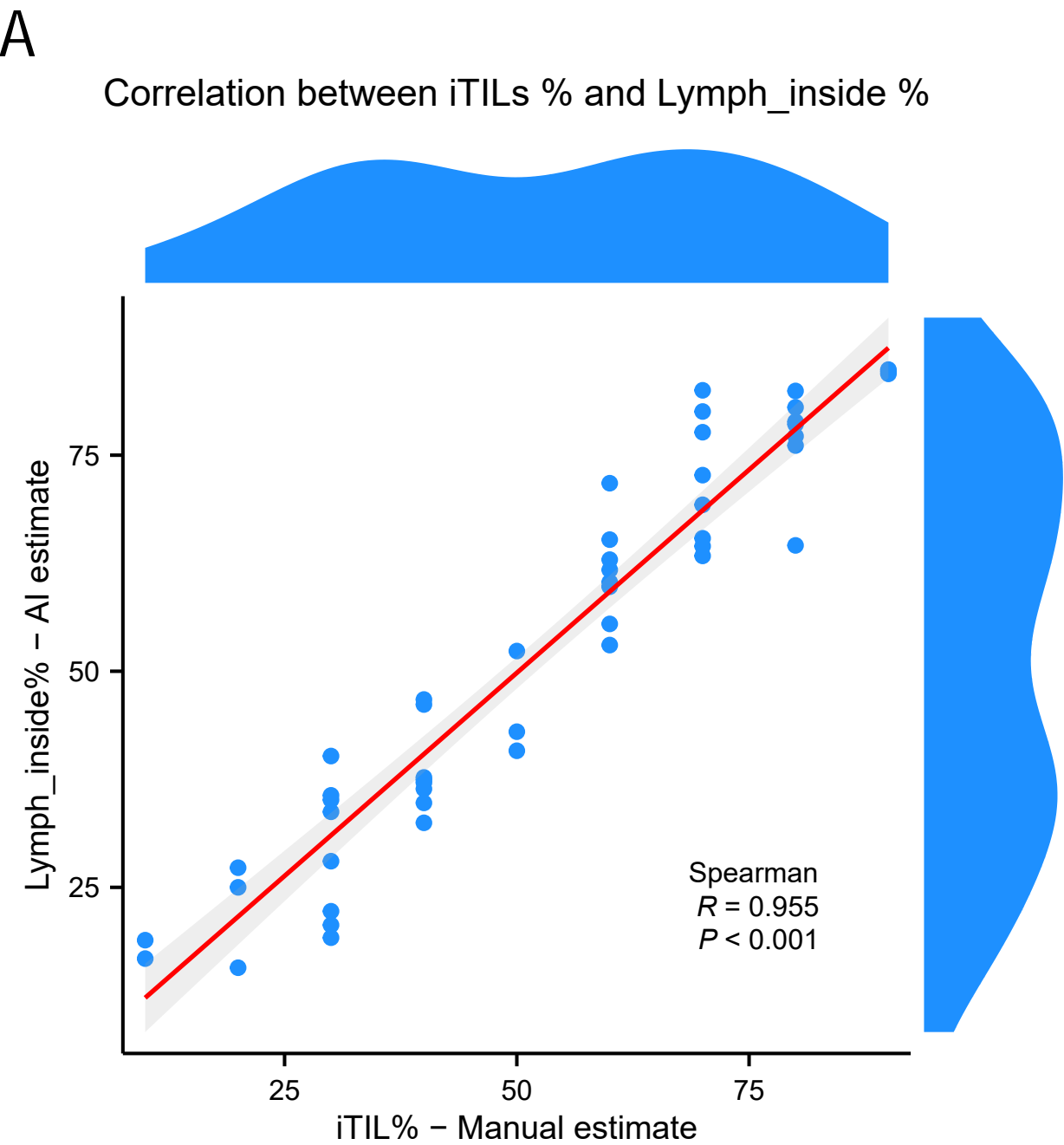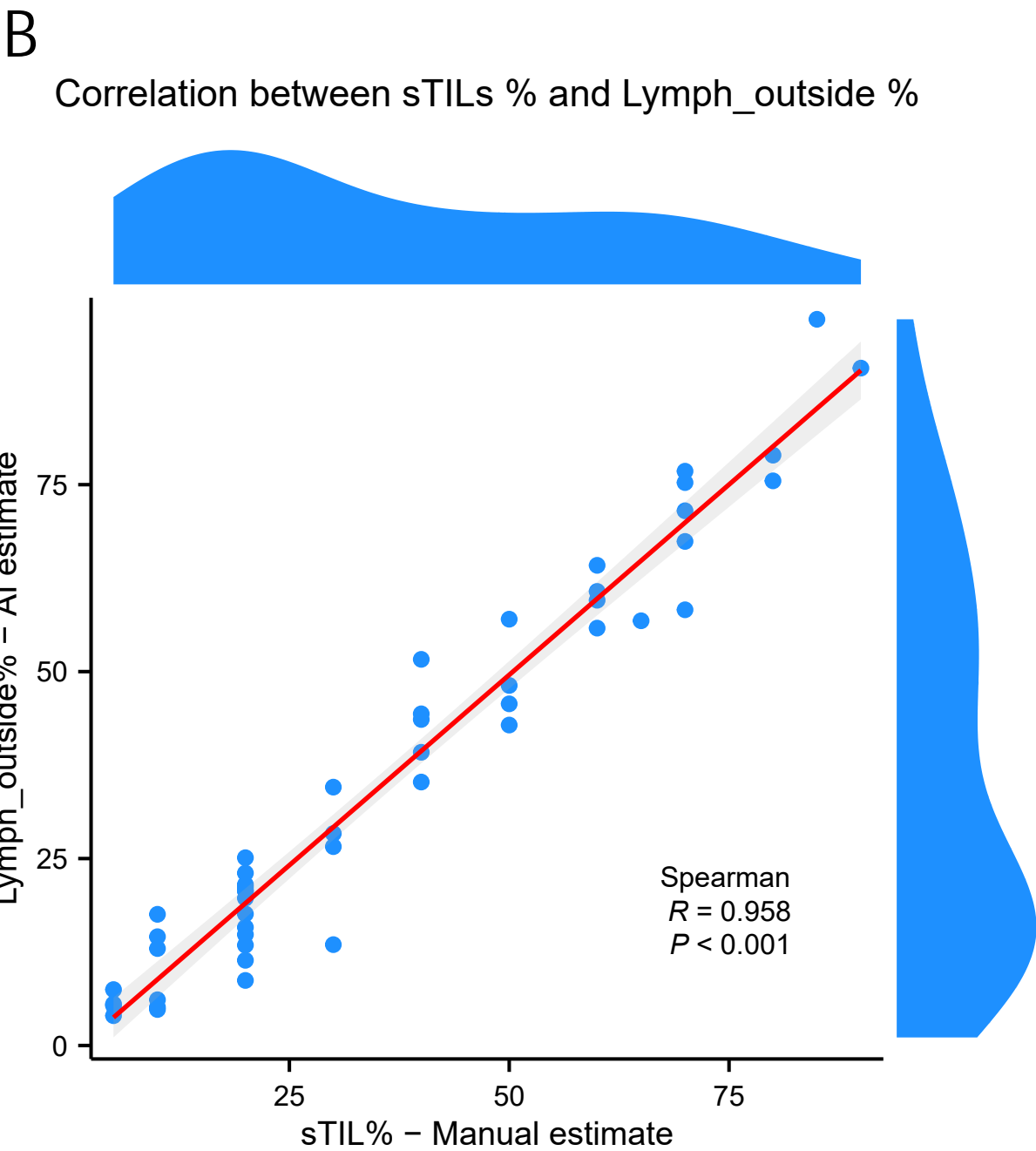

Supplement: S1 Fig — (PDF) [file pone.0328816.s001.pdf]

S2 Fig. Correlation between spatial indicators and clinical factors

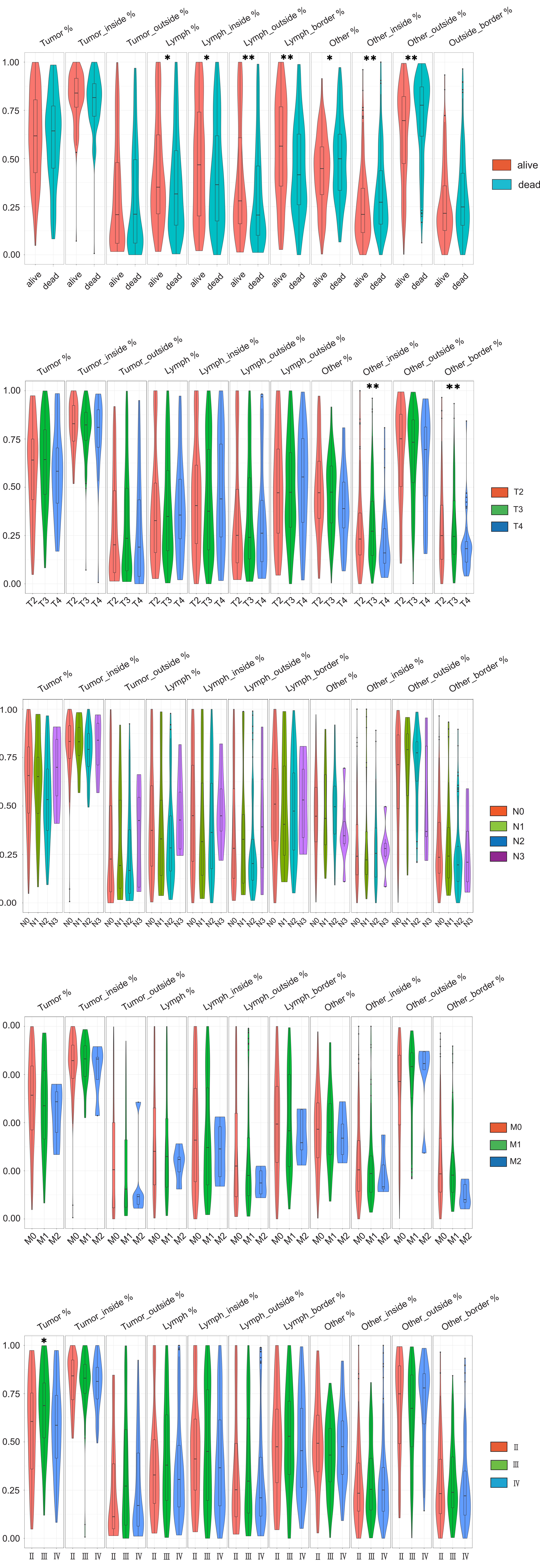

Supplement: S2 Fig — (PDF) [file pone.0328816.s002.pdf]

S3 Fig. Correlation between spatial indicators and patient survival (TCGA)

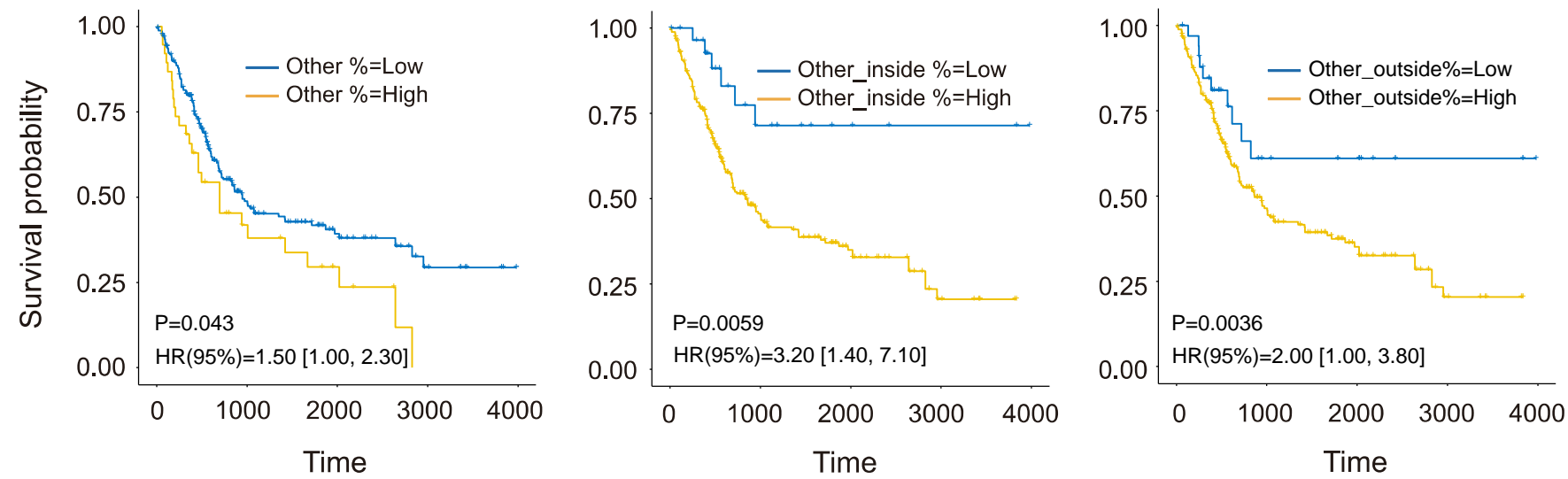

Supplement: S3 Fig — (PDF) [file pone.0328816.s003.pdf]

S4 Fig. Correlation between spatial indicators and patient survival (TMA)

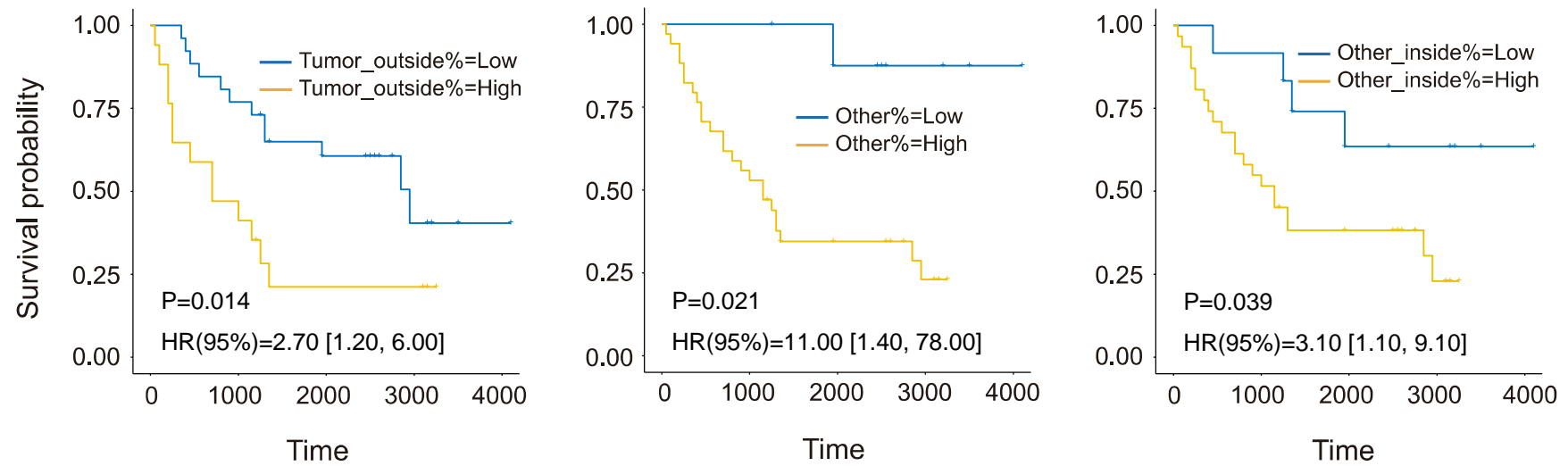

Supplement: S4 Fig — (PDF) [file pone.0328816.s004.pdf]

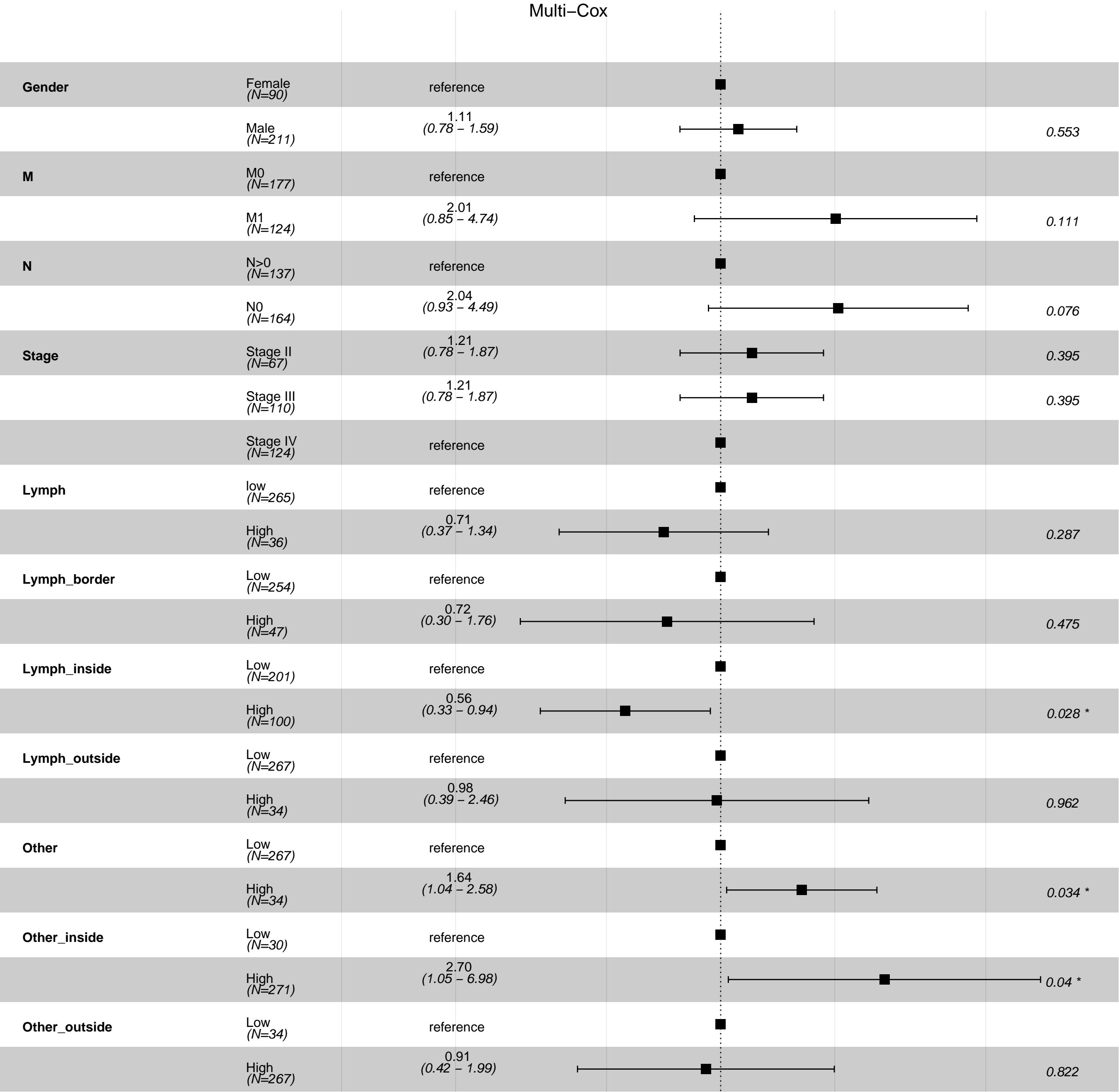

# Events: 153; Global p-value (Log-Rank): 6.  
8396e-06 AIC: 1521.61; Concordance Index: 0.65

Supplement: S3 Table — (PDF) [file pone.0328816.s007.pdf]
