# Supplementary material for: Deep learning-based spatial analysis on tumor and immune cells of pathology images predicts MIBC prognosis
Source: PLoS One. 2025 Aug 20;20(8):e0328816. doi: 10.1371/journal.pone.0328816 (PMC12367112; doi:10.1371/journal.pone.0328816)
Supplement: S1 Table — (PDF) [file pone.0328816.s005.pdf]

S1 Table. Clinical and histopathological characteristics

**Characteristic**	**TCGA** N = 301[1]	**test** N = 43[1]	**p-value**[2]
age	70 (60, 76)	72 (61, 77)	0.6
gender			0.029
female	90 (30%)	6 (14%)	
male	211 (70%)	37 (86%)	
status			0.4
Alive	148 (49%)	18 (42%)	
Dead	153 (51%)	25 (58%)	
m			<0.001
M>0	124 (41%)	5 (12%)	
M0	177 (59%)	38 (88%)	
n			<0.001
N＞0	137 (46%)	8 (19%)	
N0	164 (54%)	35 (81%)	
t			0.008
T2	90 (30%)	23 (53%)	
T3	168 (56%)	17 (40%)	
T4	43 (14%)	3 (7.0%)	
stage			<0.001
Stage II	67 (22%)	19 (44%)	
Stage III	110 (37%)	19 (44%)	
Stage IV	124 (41%)	5 (12%)	

[1] Median (Q1, Q3); n (%)

[2] Wilcoxon rank sum test; Pearson's Chi-squared test
