# Supplementary material for: Deep learning-based spatial analysis on tumor and immune cells of pathology images predicts MIBC prognosis
Source: PLoS One. 2025 Aug 20;20(8):e0328816. doi: 10.1371/journal.pone.0328816 (PMC12367112; doi:10.1371/journal.pone.0328816)
Supplement: S2 Table — (PDF) [file pone.0328816.s006.pdf]

S2 Table . Patches data of CNN training

TCGA-MIBC (50)	Tumor	Not Tumor	Lymph	Not Lymph
TCGA-01	2107	6422	1770	6759
TCGA-02	1526	8434	2147	7813
TCGA-03	6862	2681	111	9432
TCGA-04	8905	897	908	8894
TCGA-05	6685	4185	2	10868
TCGA-06	5588	860	16	6432
TCGA-07	5385	2599	1446	6538
TCGA-08	6571	2669	1360	7880
TCGA-09	11053	3074	9086	5041
TCGA-10	5160	217	2265	3112
TCGA-11	8798	6136	8516	6418
TCGA-12	9027	4999	4519	9507
TCGA-13	10297	4412	3595	11114
TCGA-14	6699	6964	3390	10273
TCGA-15	3863	9440	3349	9954
TCGA-16	5547	7698	645	12600
TCGA-17	2445	12060	3604	10901
TCGA-18	3885	5069	4043	4911
TCGA-19	11229	3014	4134	10109
TCGA-20	4663	952	2995	2620
TCGA-21	2195	12191	862	13524

TCGA-22	2653	1309	593	3369
TCGA-23	4514	4597	811	8300
TCGA-24	6478	2145	1906	6717
TCGA-25	1712	3940	1180	4472
TCGA-26	648	5378	1082	4944
TCGA-27	12816	4539	10039	7316
TCGA-28	3616	2474	654	5436
TCGA-29	7982	715	7936	761
TCGA-30	3445	6293	3680	6058
TCGA-31	4632	4108	2210	6530
TCGA-32	4358	9870	3855	10373
TCGA-33	700	2884	603	2981
TCGA-34	389	7886	652	7623
TCGA-35	10518	4651	5241	9928
TCGA-36	6170	8284	5079	9375
TCGA-37	4796	9649	5733	8712
TCGA-38	7550	5843	4377	9016
TCGA-39	6802	7737	2233	12306
TCGA-40	8518	5303	4790	9031
TCGA-41	8122	5208	5804	7526
TCGA-42	10099	5031	2282	12848
TCGA-43	1976	9048	1378	9646

TCGA-44	3575	5030	3871	4734
TCGA-45	6425	7466	5753	8138
TCGA-46	7701	3034	6871	3864
TCGA-47	8342	6349	6391	8300
TCGA-48	2605	4776	520	6861
TCGA-49	6161	1574	337	7398
TCGA-50	5560	5944	985	10519
