# Supplementary material for: Deep learning-based spatial analysis on tumor and immune cells of pathology images predicts MIBC prognosis
Source: PLoS One. 2025 Aug 20;20(8):e0328816. doi: 10.1371/journal.pone.0328816 (PMC12367112; doi:10.1371/journal.pone.0328816)
Supplement: S4 Table — (PDF) [file pone.0328816.s008.pdf]

S3 Table . Patches data of TMA

TMA-MIBC (43)	Tumor	Not Tumor	Lymph	Not Lymph
A01	83	68	29	122
A02	37	78	8	107
A03	49	118	52	115
A04	134	138	122	150
A05	107	36	10	133
A06	36	54	8	82
A07	93	54	30	117
A08	37	76	11	102
A09	41	75	11	105
A10	15	89	19	85
A11	72	137	76	133
A12	55	133	113	75
A13	32	71	36	67
A14	53	80	37	96
A15	84	141	128	97
A16	21	116	23	114
A17	80	96	55	121
A18	91	68	22	137
A19	86	101	81	106
A20	63	26	2	87
A21	54	36	2	88

A22	92	85	55	122
A23	103	87	63	127
A24	106	98	80	124
A25	119	145	114	150
A26	14	89	31	72
A27	95	115	87	123
A28	103	55	19	139
A29	42	64	41	65
A30	11	91	55	47
A31	53	99	77	75
A32	40	87	25	102
A33	9	141	52	98
A34	127	92	69	150
A35	15	97	60	52
A36	124	50	37	137
A37	105	18	2	121
A38	58	82	7	133
A39	61	52	0	113
A40	17	76	6	87
A41	115	33	7	141
A42	120	22	0	142
A43	33	110	35	108
